# High-Throughput and High-Sensitivity Biomarker Monitoring in Body Fluid by Fast LC SureQuant IS-Targeted Quantitation

**DOI:** 10.1016/j.mcpro.2024.100868

**Published:** 2024-10-22

**Authors:** Konstantinos Kalogeropoulos, Simonas Savickas, Aleksander M. Haack, Cathrine A. Larsen, Jacek Mikosiński, Erwin M. Schoof, Hans Smola, Louise Bundgaard, Ulrich auf dem Keller

**Affiliations:** 1 Department of Biotechnology and Biomedicine, Technical University of Denmark, Kgs. Lyngby, Denmark; 2 Poradnia Chorób Naczyń Obwodowych "MIKOMED", Łódź, Poland; 3 Paul Hartmann AG, Heidenheim, Germany

**Keywords:** targeted proteomics, SureQuant, internal standard PRM, high throughput, biomarker, body fluid, wound healing, wound fluid, clinical proteomics

## Abstract

Targeted proteomics methods have been greatly improved and refined over the last decade and are becoming increasingly the method of choice in protein and peptide quantitative assays. Despite the tremendous progress, targeted proteomics assays still suffer from inadequate sensitivity for lower abundant proteins and throughput, especially in complex biological samples. These attributes are essential for establishing targeted proteomics methods at the forefront of clinical use. Here, we report an assay utilizing the SureQuant internal standard–triggered targeted method on a latest generation mass spectrometer coupled with an EvoSep One liquid chromatography platform, which displays high sensitivity and a high throughput of 100 samples per day. We demonstrate the robustness of this method by quantifying proteins spanning six orders of magnitude in human wound fluid exudates, a biological fluid that exhibits sample complexity and composition similar to plasma. Among the targets quantified were low-abundance proteins such at tumor necrosis factor A and interleukin 1-β, highlighting the value of this method in the quantification of trace amounts of invaluable biomarkers that were until recently hardly accessible by targeted proteomics methods. Taken together, this method extends the toolkit of targeted proteomics assays and will help to drive forward mass spectrometry–based proteomics biomarker quantification.

## Highlights

• Quantification across six orders of magnitude, including biomarkers TNFA and IL1B.
• Processes up to 100 samples per day using EvoSep One chromatography.
• Suitable for complex samples with consistent results across multiple samples.

## Graphical Abstract

**Figure:**
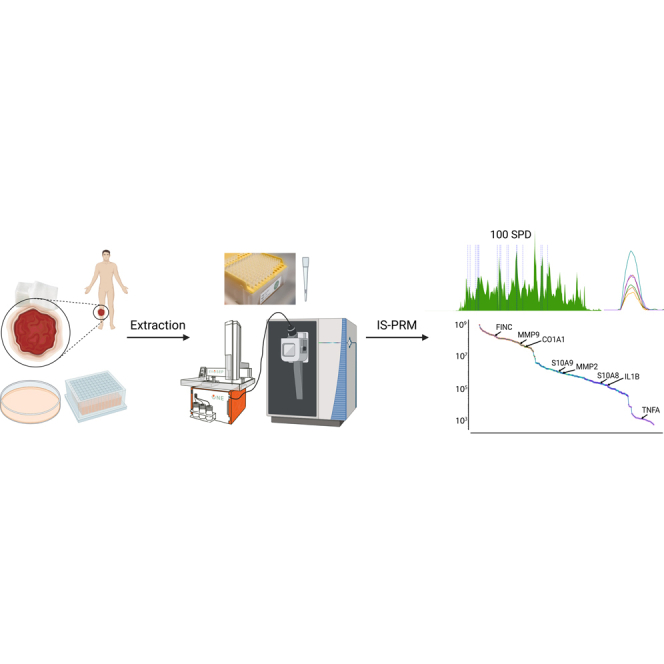

## In Brief

Targeted proteomics has advanced significantly but still faces challenges in detecting low-abundance proteins and providing high throughput in complex biological samples. This study presents an optimized and robust method combining the SureQuant internal standard–triggered approach with EvoSep One. The assay was tested on human wound fluids, quantifying biomarkers like TNFA and IL1B. The results highlight this method's potential for precise and sensitive biomarker detection in clinical and large-scale studies.

Analysis of disease-related protein biomarker panels in various tissues and body fluids is essential for reliable clinical diagnostics and evaluation of treatment regimens, as well as in pharmaceutical research and development. This calls for a reproducible, robust, and sensitive high-throughput workflow. Over the past decade, parallel reaction monitoring (PRM) has been developed into a significant and very reliable mass spectrometry (MS)-based proteomics method for simultaneous quantification of multiple proteins in complex biological samples (1). PRM relies on selective and sensitive quantification of endogenous peptides and mostly uses quadrupole-orbitrap mass spectrometers (2, 3). However, one of the major challenges in traditional PRM analysis is the requirement to accommodate more peptides into a narrow retention time window with an increasing number of target peptides and a concurrent decrease in sensitivity with higher degrees of multiplexing. This limitation is particularly critical in high-throughput studies where large numbers of samples are to be analyzed. Although shorter chromatographic gradients in these experiments are intuitively highly desirable, they are difficult to implement, since more peptide precursors are concurrently eluted from the analytical column, impeding detection and limiting sensitivity. This affects the overall performance of the method in large-scale studies (4).

To overcome limitations of pre-defined retention time windows, the PRM method was modified to include stable isotopically labeled peptides as internal standards (IS) to trigger the dynamic acquisition of the endogenous peptides in real-time. In this method, the detection of an IS peptide triggers a switch from a watch mode to a quantitative mode where the IS and endogenous peptides are measured. The time window for acquisition in the quantitative mode is set to match the actual elution profile of the peptides, ensuring a more effective use of the instrument time (5).

Recently, the IS-PRM acquisition has been further refined to maximize efficacy of the method (6). This new method termed SureQuant takes advantage of enhanced capabilities of the latest generation of orbitrap instruments. In this refined acquisition, the watch mode interrogates information from both the MS1 and MS2 data to profile the elution of the IS. Instead of matching a spectral library, the identification of an IS triggers a low resolution MS2 and if the fragments match a defined number of transitions specified in the method, the instrument triggers a high resolution, high sensitivity MS2 scan for the target peptide (7). This dynamic acquisition mode ensures a higher number of successful scans than traditional PRM, allowing analysis of more targets with consistently higher sensitivity and less total instrument time. As an example, the SureQuant acquisition approach has been successfully used to quantify more than 500 endogenous peptides in human nondepleted plasma (8).

The current depth of proteome coverage in proteomics studies is not only based on technological advances of mass spectrometers but also on the optimization of sample preparation and fractionation steps prior to MS analysis (9). Systems integrating multiple pre-analysis steps are continuously developed and refined. One example is the EvoSep One system, which combines sample clean-up with a fast and efficient chromatographic separation without compromising the sensitivity and robustness known from nano-liquid chromatography (LC) systems. This system uses a low-pressure pump to elute the analyte along with the elution gradient from the clean-up cartridge into a capillary loop and then a single high-pressure pump to apply the sample to the analytical column. This abolishes the need to form a gradient at high pressure and the gain is a reduced idle time between injections. The EvoSep One concept has proved very promising for high-throughput proteomics (10).

In this study, we combined the sensitivity of SureQuant acquisition on a ThermoFisher Scientific Orbitrap Exploris 480 quadrupole-orbitrap instrument with the speed of EvoSep chromatography to devise a workflow for high-throughput and high-sensitivity biomarker monitoring in complex biological matrices. We showcase the power of the method by time-resolved monitoring of wound biomarkers spanning a concentration range of six orders of magnitude in nondepleted clinical wound exudates.

## Experimental Procedures

### Wound Exudate Preparation

#### Wound Dressings

The clinical study was planned and managed by Paul Hartmann AG (Heidenheim). The study was approved by the Bioethics Committee at the Silesian Medical Chamber, Katowice, Poland (Opinion No. 35/2018) and registered with the German Clinical Trials Register (DRKS00015832). It was conducted in accordance with the Declaration of Helsinki and laws and regulations in Poland. All patients provided written informed consent prior to entry into the study. The wound fluid was extracted from wound dressings from a cohort of six patients with venous leg ulcers (Supplemental Table S1). The patients were enrolled in the study for a total of seven visits with approximately 14-day intervals in between. At each visit, the patient and wound was examined, and the ulcer dressing was changed. At the first visit, the wound was treated with Medicomp for 2 h. Subsequently, a two-layer bandage compression therapy (PütterPro2) along with a Hydroclean wound dressing was applied. The Hydroclean wound dressing was removed during the second visit, serving as the sample containing the wound exudate for visit 2. A new Hydroclean dressing was used to cover the wound, and this procedure was repeated until visit 7, resulting in 1 Medicomp and 6 Hydroclean dressings containing wound exudates from each chronic wound patient. Dressings were stored at the clinical site at −20 °C and shipped on dry ice. Upon arrival, dressings were stored at −80 °C until further processing.

#### Extraction of Wound Fluids from Wound Dressings

A buffer solution consisting of 50 mM 4-(2-hydroxyethyl)-1-piperazineethanesulfonic acid (Hepes), 150 mM sodium chloride (NaCl), 10 mM ethylenediaminetetraacetic acid was used to incubate the dressings for protein extraction. A protease inhibitor cocktail (cOmplete, Mini, EDTA-free Protease Inhibitor Cocktail, 04693159001, Roche) was added to the buffer solution according to the manufacturer instructions. For Hydroclean wound dressings, the inner polyacrylate-based layer was removed from the outer layers, using scissors to cut around the edge of the dressing. The inner layer was placed on a Petri dish, and buffer solution was applied on the polyacrylate, at a ratio of 500 μl/cm^2^. For Medicomp dressings, the dressing was transferred to a 15 ml falcon tube, and buffer solution was added until the dressing was fully immersed. The dressings were incubated on ice for a minimum of 2 h on a rocking platform. Following incubation, Medicomp dressings were placed in a 20/50 ml syringe, and the absorbed liquid was squished through. Hydroclean dressings polymer material is prone to disruption, so the material was directly placed in a cell strainer and drained using a mortar. The liquid phases extracted for both dressing types were passed through a 40 μm cell strainer (Fisherbrand Sterile Cell Strainers, 22-363-547, Thermo Fisher Scientific) in order to separate polymer residues from the solution. Cell debris and remaining particles were removed by centrifugation (3 × 15 min, 4000*g*, 4 °C). The samples were desalted and concentrated by ultrafiltration using 3 kDa molecular weight cutoff concentrators (Amicon Ultra-15 Centrifugal Filter Unit, UFC9003, Millipore, Merck), and the final protein concentration was determined with a NanoDrop One/OneC Microvolume UV-Vis Spectrophotometer (Thermo Fisher Scientific). Samples were stored at −80 °C until further processing.

#### Sample Preparation for MS Analysis

One hundred micrograms of protein were diluted in 2.5 M guanidine hydrochloride (GuHCl), 100 mM Hepes pH 7.8 solution to a final concentration of 500 ng/μl. Cysteines were reduced and alkylated by the addition of tris(2-carboxyethyl)phosphine to a final concentration of 1.5 mM, incubated for 60 min at 65 °C, and chloroacetamide to a final concentration of 5 mM followed by incubation at 65 °C for 30 min. Afterwards, samples were diluted with 100 mM Hepes pH 7.8, in order to reach a final concentration of 0.5 M GuHCl. Trypsin was added to the samples in a final ratio of 1:50 (protease:protein w/w) and incubated for 16 h at 37 °C, 600 rpm in an Eppendorf ThermoMixer C. Reaction was stopped with 10% TFA (v/v), for a final concentration of 1% TFA v/v. The peptide concentration was measured with a NanoDrop One/OneC Microvolume UV-Vis Spectrophotometer (Thermo Fisher Scientific).

### SureQuant Assay Design

#### Selection of Peptides for Targeted Analysis

We included a total of nine proteins central in the wound-healing pathophysiology covering a broad dynamic range of concentrations in wound exudates: matrix metalloproteinase 2, matrix metalloproteinase 9, neutrophil elastase (ELNE), interleukin-1 beta, tumor necrosis factor-alpha (TNFA), S100A8, S100A9, collagen 1 (COL1A1), and fibronectin. Based on data-dependent acquisition (DDA) shotgun analysis survey runs and information from ProteomicsDB (11, 12) and PeptideAtlas (13, 14), three to seven peptides per protein were selected for assay suitability and validation. For peptides selected from databases, the most frequently observed peptides that were identified in MS experiments with high confidence and satisfying a number of predetermined criteria (no methionine, length between 8 and 21 amino acid residues, C-terminal arginine or lysine, no missed cleavages) wherever possible were chosen for subsequent experiments. In proteins undergoing maturation due to proteolytic processing, preference was given to peptides matching the processed mature protein. The heavy isotopic arginine or lysine-labeled peptides for IS-PRM analysis were ordered from JPT Peptide Technologies. The quantotypic properties of the selected peptides were tested, and the two peptides with best intensity and elution profiles for each protein were selected for the final targeted analysis (Supplemental Table S2).

### LC-MS/MS Analysis

#### EvoTip Sample Clean-Up and Loading

EvoTip columns were conditioned with 100% isopropanol for 1 min, washed once with 50 μl buffer B (80% acetonitrile, 0.1% TFA) by centrifugation for 30 s, 1400 g. Activation with 100% isopropanol was repeated followed by equilibration with 50 μl buffer A (0.1% formic acid (FA)) and centrifugation for 10 s, 1400*g*, leaving approximately 30 μl of buffer A on the EvoTip. The sample was loaded onto the EvoTip, followed by centrifugation for 40 s at 1400*g*. The loaded peptides were washed two times with 200 μl buffer A using a centrifugation step for 40 s, 1400*g* each. The washed peptides were kept wet by applying 250 μl of buffer A on top of the EvoTip and centrifugation for 10 s at 1400*g*.

#### Reverse Phase Liquid Chromatography

The peptides on the EvoTips were separated on an Evosep One chromatography system using a 4 cm × 75 μm, PepMap RSLC analytical column, packed with 2 μm C18 beads. Columns were washed and equilibrated before each run and peptides separated from the stationary phase over 11.5 min or 22 min according to the manufacturer standard method 100SPD or 60SPD, respectively. The final optimized assay made use of the 100SPD method as defined by the manufacturer, with analytical column equilibration and washing at 2 μl/min, active gradient length of 11.5 min, cycle time of 14.4 min, and flow rate of 1.5 μl/min. Peptides were eluted from the column with solvent A (0.1% formic acid in water) and gradually increasing concentration of solvent B (100% acetonitrile).

#### Shotgun Mass Spectrometry Analysis for Synthetic Peptide Library

The spectral library was generated with an EASY-nLC 1200 (Thermo Fisher Scientific), using an EasySpray column (Thermo Fisher Scientific, cat. no. ES903, 2 μm particle size, 75 μm diameter, 50 cm length) and a 100 min gradient (6% buffer B at 0 min, increasing to 23% at 55 min, 38% at 80 min, 60% at 85 min, 95% at 88 min until the end of the gradient). An Orbitrap Exploris 480 mass spectrometer (Thermo Fisher Scientific) equipped with a FAIMSpro ion mobility device (ThermoFisher Scientific) operated in DDA mode was used for data acquisition. A sample volume corresponding to 1000 ng of peptides and 0.8 pmol of heavy peptides was injected (Supplemental Table S3). The FAIMSpro device was set to a single compensation voltage (CV) of −50. MS1 resolution was set to 60,000, automatic gain control (AGC) target set to 300%, maximum injection time set to automatic, scan range 375 to 1500 m/z, selecting the top 28 MS1 ions for MS2 analysis. MS2 scans used 15,000 resolution, AGC target of 75%, maximum injection time 60 ms, and isolation window of 1.6 m/z at normalized collision energy of 28.

#### SureQuant Analysis

Gradient optimization was carried out with 11.5 min (100SPD gradient) or 22 min (60SPD gradient) gradients, on an Orbitrap Exploris 480 mass spectrometer operated in SureQuant mode. SureQuant analysis was performed on an Orbitrap Exploris 480 mass spectrometer (Thermo Fisher Scientific) operated in SureQuant mode for 11.5 min (100SPD gradient). A sample volume corresponding to 1000 ng of sample peptides and 0.8 pmol of heavy peptides was injected. MS1 resolution of 120,000, AGC target of 300%, 50 ms injection time, and cycle time of 2 s. This was followed by heavy peptide precursors and fragment recognition from the list (Supplemental Tables S4 and S5), at a resolution of 7,500, high collision dissociation energy at 28, AGC target of 1000%, and a 10 ms injection time. On the fly product, ion trigger had to equal at least three product ions to initiate an offset scan (Supplemental Table S6) at a resolution of 60,000, high collision dissociation collision energy at 28, AGC target of 1000%, and 250 ms injection time in centroided mode. The same method was used with FAIMS on and a CV of −50 in the comparison between the two setups.

### MS Data Analysis

#### Shotgun MS Data Analysis

Data analysis was performed using Proteome Discoverer 2.2 (Thermo Fisher Scientific). MS spectra were extracted from raw data files and searched against a complete *Homo sapiens* database from the UniProt Database (TaxID 9606, 42,252 SwissProt entries, November 25, 2017). The database was concatenated with a list of all protein sequences in reversed order. Searches were run with 10 ppm precursor ion tolerance for total protein level identification and 0.02 Da fragment ion tolerance using SEQUEST search algorithm and Percolator for false discovery rate (FDR) filtering. Carbamidomethylation of cysteine residues (+57.021 Da), heavy lysine, and arginine amino acids (+8.014 and + 10.008 Da) were set as static modifications, while oxidation (+15.995 Da) was set as a variable modification. Peptide-spectrum matches were set to not exceed 1% FDR. Filtered peptide-spectrum matchs were further filtered for peptide and protein-level FDR of 1%.

#### SureQuant Data Analysis

To analyze SureQuant data, we used Skyline 20.1 from the MacCoss lab (15). Target peptides were imported after setting filter parameters at MS1 orbitrap to centroided, a mass accuracy of 10 ppm and MS/MS DIA centroided filtering at 20 ppm, with fixed isolation scheme of 0.007 m/z. All precursor masses had at least three transitions. Endogenous group of peptides had a fixed modification of carbamidomethylation of cysteine residues (+57.021 Da); synthetic peptides had additional static modifications of heavy lysine and arginine amino acids (+8.014 and + 10.008 Da). The peptide transition peaks and their integration boundaries were manually revised and adjusted based on the heavy peptide retention times. Peak areas for the peptide fragments ions were exported from Skyline and the total area fragment column of the report for each precursor was used for quantification. Further processing and analysis were performed with custom-made Python scripts (Python version 3.6), available upon request from the corresponding author. The peptide fragment areas were normalized based on the total ion current chromatogram area, extracted from the raw data using the RawTools package (https://github.com/kevinkovalchik/RawTools). Peptide values from each protein were averaged to get protein quantification values. The protein values were rescaled on a 0 to 100 scale separately for each protein.

### Immunoassay Experiments

#### Multiplex Immunoassay Analysis

For the multiplex bead-based immunoassay analysis, we used a custom made Human Magnetic Luminex Assay LXSAHM (R&D systems) according to manufacturer’s instructions (https://www.rndsystems.com/products/human-magnetic-luminex-assay_lxsahm). Reference standards for all analytes except ELNE were provided in the assay kit, and the assay was thoroughly validated by the manufacturer. An additional specificity validation was performed by eight different exclusion tests, where seven out of the eight recombinant biomarkers were mixed and analyzed. Final target concentrations for the recombinant biomarkers in the test were aimed at the middle of the 7-point standard curves (Table 1). The sample concentration was adjusted to 50 μg/μl using 50 mM Hepes. Initial dilution tests were performed on three patient samples at 1:2, 1:25, 1:50, 1:200, 1:4000, and 1:6000 dilutions with kit-provided dilution buffer. After evaluation, each of the patient samples were diluted 1:50, 1:500, and 1:10,000 and analyzed. All standards were measured in triplicates, and all samples were measured in duplicates. The plates were analyzed on a Luminex MAGPIX (Luminex corporation).

**Table 1:** Target concentrations for recombinant biomarkers in specificity test

Biomarker	Target concentration (pg/ml)
Collagen I alpha 1 (6220-CL-020)^a^	260
Fibronectin (1918-FN-02M)^a^	59,100
IL1B (201-LB005/CF)^a^	316
MMP-2 (in house, unknown origin)	5416
MMP-9 (in house, unknown origin)	2108
S100A8 (9876-S8)^a^	1205
S100A9 (9254-S9)^a^	426
TNFA (210-TA-005/CF)^a^	143

a From Biotechne, Abingdon, UK.

#### Multiplex Immunoassay Data Analysis

Raw data was analyzed using xponent software (version 4.2.1324.0, Luminex corporation). Validation of data was performed as follows: If highest or lowest standards were <10% higher or lower, respectively, from the nearest standard based on net median fluorescence intensity (MFI) minus background, then they were removed due to flattening of the curve. If the percentage recovery of a standard, based on the five parameter logistic regression made by Xponent, was outside 80 to 120%, then the standard was removed due to poor model fit. If the percent coefficient of variance (% CV) of the net MFI was more than 10%, then the standard was removed due to poor replication. For the samples, net MFI values above or below the standard curve average net MFI was discarded. Moreover, sample measurements were removed if percent CV was more than 10%. Finally, the absolute concentration of the different biomarkers in each sample was calculated based on the standard curve and the dilution factor.

### Experimental Design and Statistical Rationale

Quantification relied on five transitions from two peptides for each protein in accordance with the HUPO guidelines for high confidence PRM/SRM assay protein quantitation (16) and following guidelines for Tier 3 assays (17). The absolute protein concentrations and dynamic range measurements by Luminex assays were based on the analysis of a total of 42 biological replicates.

## Results and Discussion

In this work, we introduce an optimized internal standard–based SureQuant PRM workflow that we apply in a case study of biomarker quantification using a total of 42 human wound exudate samples. We demonstrate the robustness and sensitivity of the acquisition method by targeting peptides, representing both highly and lowly abundant proteins in a highly complex matrix. This targeted proteomics workflow exhibited high sensitivity, reproducibility, and robustness.

### Experimental Workflow and Design

**Fig. 1:**
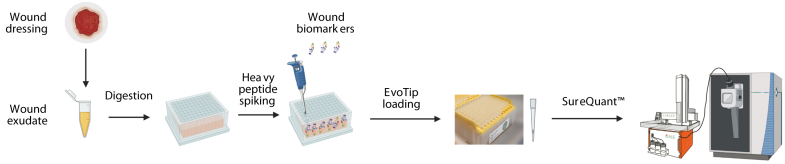
**Sample preparation and analysis workflow.** Wound exudates were extracted from dressings and prepared for analysis with no depletion of high-abundance proteins. Samples were digested on 96-well plates. After digestion, heavy peptides for wound biomarkers were spiked into the samples and the mixture loaded on EvoTip trap columns. Finally, columns were placed on an Evosep One platform and analyzed on a ThermoFisher Scientific Orbitrap Exploris 480 mass spectrometer.

Our newly devised targeted proteomics workflow follows the general principle of SureQuant analysis with significant modifications by integration of the EvoSep One platform for fast LC on a ThermoFisher Scientific Orbitrap Exploris 480 instrument (Fig. 1). In our case study, we used trypsin as digestion protease and analyzed nine biomarkers central in wound pathophysiology known to span a large dynamic range in concentrations in wound fluids (18, 19). Thereby, we targeted two peptides per protein that had been selected either from DDA shotgun analyses or with the help of ProteomicsDB (20) or PeptideAtlas (13), respectively, and following the HUPO guidelines for high confidence PRM/SRM assay protein quantitation (16) and criteria as outlined in Experimental Procedures.

### Dynamic Range of Selected Wound Fluid Biomarkers

**Fig. 2:**
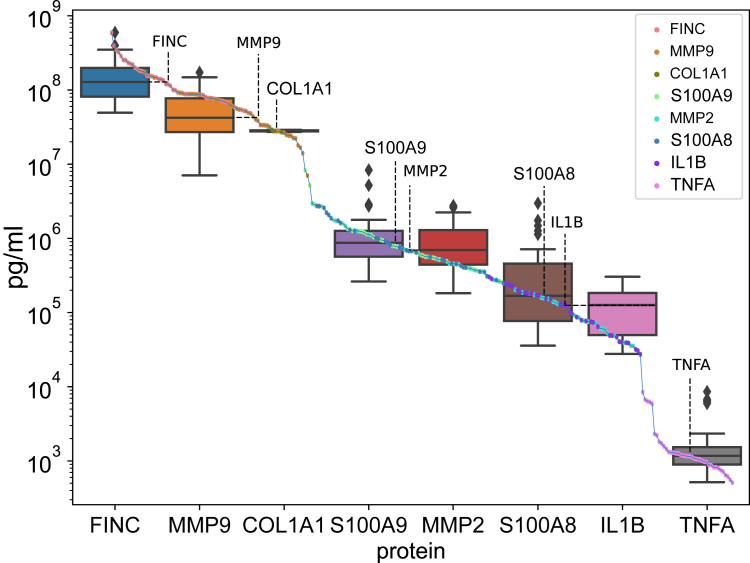
**Absolute quantification of proteins of the biomarker panel in wound exudates.** Protein concentrations were measured using a custom-made Luminex assay in all 42 patient samples. The measurement distribution for each protein is given as boxplots displaying the median measurement and outlier measurements as whiskers (75th quantile). Measurements for all proteins analyzed are also given as a run plot indicating the full measurement range of the MS assay.

To evaluate the power of our workflow, we first determined the actual concentration range of the selected wound biomarkers in the 42 patient samples included in this study. For this, we used a validated custom-made bead-based multiplexed immunoassay with the Luminex technology upon optimization of sample dilutions for each protein biomarker of interest (Supplemental Table S7). As anticipated from literature reports on protein amounts in similar samples (18, 19), absolute concentrations of the selected proteins in extracted wound exudates spanned six orders of magnitude (Fig. 2). At the extremes, fibronectin had a median concentration value of 128 μg/ml, while the median concentration across all samples for TNFA was 1171 pg/ml (Supplemental Table S8). Thus, the samples and selected biomarker proteins used to test specificity and sensitivity of our targeted proteomics workflow presented a highly dynamic concentration range in a complex sample matrix.

### Adaptation of SureQuant to Short LC Gradients

**Fig. 3:**
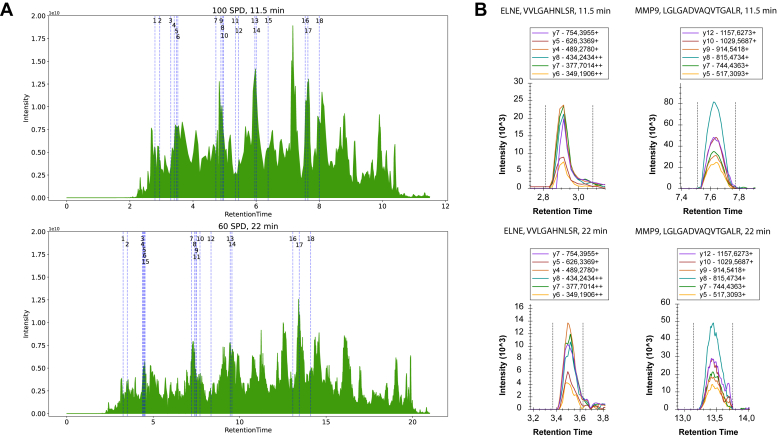
**Assessment of EvoSep One LC gradients.***A*, selected example total ion current chromatograms for 11.5 (*top*) and 22 min gradients (*bottom*). *Dashed lines* indicate the peak of the elution window for peptide precursors and their transitions. *B*, selected example transition plots for endogenous peptides of neutrophil elastase and MMP9, using 11.5 (*top*) and 22 min gradients (*bottom*).

Since throughput is critical for biomarker studies with larger patient cohorts, we next sought to shorten LC gradients as major time-limiting step in sequential-targeted proteomics analyses of many samples. This became possible with help of the EvoSep One system that provides optimized workflows for processing of either 60 or 100 SPD, resulting in 22- and 11.5-min gradients, respectively. Despite inherent compression of total ion currents from shorter gradients, the 18 heavy spike-in peptides (selected to identify and relatively quantify the nine biomarkers in this study, Supplemental Tables S9 and S10) were sufficiently resolved along the elution range both in 60 and 100 SPD setups (Fig. 3*A*). Moreover, shortening LC gradients did neither affect the number of reliably monitored transitions nor peak areas but in many cases even resulted in sharper peak groups with respective smaller peak widths (Fig. 3*B*). Applicability of fast LC has been demonstrated for standard PRM assays (21) but not yet reported in the literature for IS-PRM and SureQuant. Hence, our workflow demonstrates the potential to increase throughput of targeted analyses of proteins spanning a large dynamic range in a complex biological matrix.

**Fig. 4:**
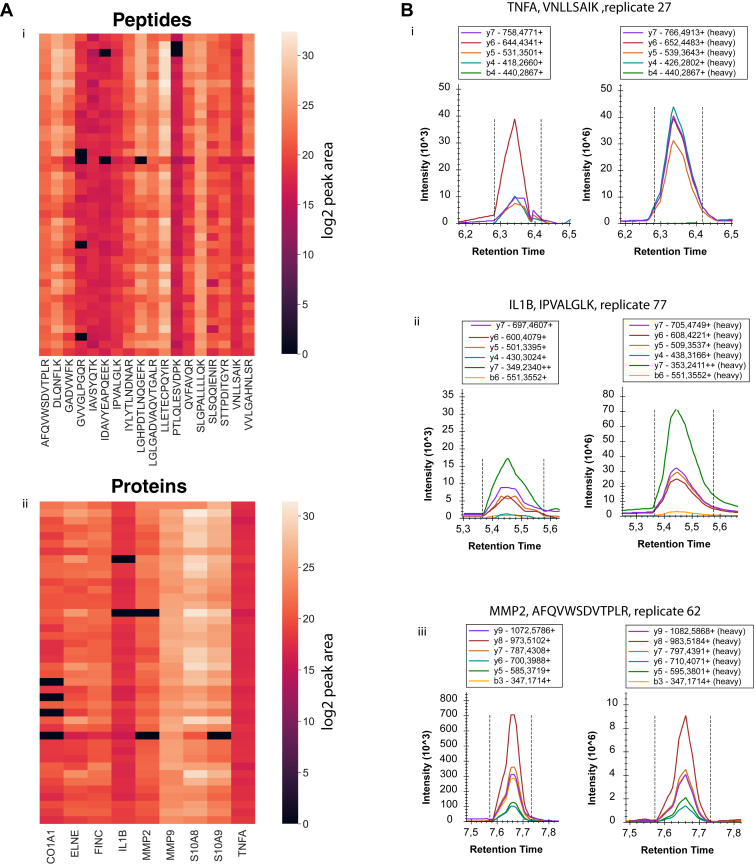
**Data completeness and peak area intensity analysis and examples of elution profiles and transitions for selected peptides.***A*, heatmaps showing log2 peak area for proteins and peptides. i: Log2 transformed values of integrated peaks of ion transitions for each peptide precursor. ii: Log2 transformed protein values of averaged integrated peaks for precursor peptides. *B*, selected examples of measured precursor ion transitions for i: Peptide VNLLSAIK, TNFα, ii: Peptide IPVALGLK, IL1β, iii: Peptide AFQVWSDVTPLR, MMP2.

The analytes measured were detectable and quantifiable in all but few of the clinical samples (Fig. 4*A*). To our knowledge, this is the first example of quantification of the lowly abundant cytokines interleukin-1 beta and TNFA by targeted proteomics in human samples with complexity and composition resembling blood plasma (Fig. 4*B*). However, it has to be noted that the samples used in this study likely showed elevated levels of the two cytokines, in most cases more than 50-fold over baseline levels, due to the chronic inflammatory wound environment. Near native concentrations of low-abundance biomarkers might not be reliably measurable yet but could potentially be realized in the near future with further developments in workflow and instrumentation.

We compared the detection and sensitivity of our workflow with or without FAIMS coupled to our LC-MS/MS setup. We observed decreased precursor identifications and diminished signal intensity when the samples were measured with FAIMS (Supplemental Fig. S1). However, we used a single compensation voltage of −50 and did not optimize CVs for each peptide of our panel, which might increase sensitivity with FAIMS-PRM. We also compared the quantification of the analytes measured between the immunoassay and MS-based monitoring performed. In general, the two methods show positive correlation in the relative abundance of our protein panel. Both technologies capture relative changes and exhibit similar patterns in protein abundance over time in the patient wound fluids (Supplemental Fig. S2). We observed a discrepancy in TNFA levels, which could potentially be explained by high technical variability due to low abundance and interfering ions in the SureQuant analysis.

The high levels of these markers in the initial stages of patient treatment highlight a highly inflammatory and degrading environment, which is hindering wound-healing progression in impaired wounds. This effect seems to be attenuated in later timepoints where samples were collected, indicating resolution of the inflammatory phase and transition to a healing trajectory.

### High-Throughput Analysis

**Fig. 5:**
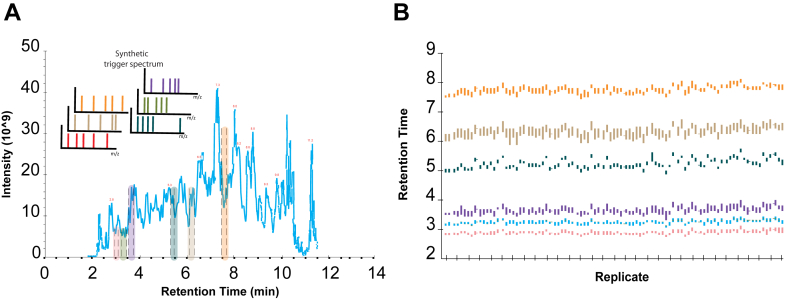
**Retention time of peptides used in the targeted experiments.***A*, example of a sample chromatogram with the gradient used for all targeted experiments of the study and overview of elution windows for a set of six precursors. *B*, retention time of selected precursors in a stress test analysis of 365 samples (24), with 91 shown in the figure.

With an increased sensitivity, we wanted to ensure the reproducibility and throughput of these assays. This was addressed by using the EvoSep One system (22) together with SureQuant acquisition. Optimization trial runs determined the chromatographic gradient of 11.5 min to exhibit the highest benefits in terms of sensitivity, quantification reliability, and high throughput analysis. The combination of the two systems retained the measurement window within 30 s and peak detection resulted in most cases in coverage of the entire area under the curve of the endogenous peptides, with an average of seven data points per peak for all peptides (Fig. 5*A*). The retention time windows for all peptides showed very high consistency even after a stress test of nonstop analysis of 365 samples, with mean SD of less than 7 s across the 18 quantified peptides and maximum SD of 10 s (Fig. 5*B*). By harnessing the high sensitivity of the ThermoFisher Scientific Orbitrap Exploris 480 mass spectrometer and the standardized 100SPD method from the EvoSep One system, we were able to identify and quantify the target proteins.

## Conclusions

This study demonstrates that the SureQuant IS-PRM method presents an excellent strategy for targeted, quantitative proteomics. The method provides the opportunity for high-throughput analysis of peptides in a wide dynamic range without sacrificing measurement sensitivity. The dependence on heavy peptides for the detection of the endogenous peptides bears some risks and requires cycle times to be optimized in order to preserve the initial peptide elution profile. However, the addition of heavy peptides in the matrix does not seem to affect sample measurement. The heavy peptides retain the same chromatographic properties as their native counterparts (5). In addition, it still seems to be a considerably better alternative to precursor monitoring scheduling windows, which is the strategy used in standard PRM experiments. This holds especially true in high-throughput experiments that span several days of continuous instrument running time, where retention time shifts are frequently observed due to multiple, continuous injections in the same analytical column (10, 23). The combination of the EvoSep HPLC system and the SureQuant method present an excellent example of reliable high throughput targeted experiments monitoring multiple precursors, with the capability of running 100 SPD without sacrificing sensitivity.

We believe this method is translatable to other complex biofluids such as human plasma and cerebrospinal fluid with limited optimization. However, despite the complexity of our samples, sensitivity and detection of analytes in such low concentration might be hindered in other biofluids, due to higher complexity and dynamic range, as well as different protein composition. This method could be effectively applied to wound exudates, enabling biomarker monitoring for prognosis, evaluation of wound healing progression, or clinical intervention decision-making.

## Data availability

All mass spectrometry–based proteomics data discussed in this study have been deposited at PanoramaWeb (24) (https://panoramaweb.org/surequant_method.url) (e-mail: panorama+reviewer111@proteinms.net; password: dZEWyxvy) with the dataset identifier PXD032305 (http://proteomecentral.proteomexchange.org/cgi/GetDataset?ID=PXD032305).

## Conflicts of Interest

H. S. is a full-time employee of HARTMANN. The other authors declare no competing interests.

## Supplementary Data

Suppl_figure_1 *A*, examples of TIC intensities between SureQuant and SureQuant with FAIMS. Addition of FAIMS during MS analysis results in reduced signal for the analytes measured. ***B***, quantification values for the peptides monitored across SureQuan and SureQuant FAIMS analysis. Non detected or filtered precursors are indicated with black boxes.

Suppl_figure_2 Comparison of quantification between Luminex and SureQuant measurements, for the 8 proteins measured. Collagen 1 A1 chain was not quantified in most samples with the Luminex technology due to values outside the standard curve. Values are normalized to the highest quantity observed. In SureQuant quantification, peptide intensitites are summed for protein quantification values.
