# Supplementary figures and images for: High-Throughput and High-Sensitivity Biomarker Monitoring in Body Fluid by Fast LC SureQuant IS-Targeted Quantitation
Source: Mol Cell Proteomics. 2024 Oct 22;23(12):100868. doi: 10.1016/j.mcpro.2024.100868 (PMC11609441; doi:10.1016/j.mcpro.2024.100868)

A

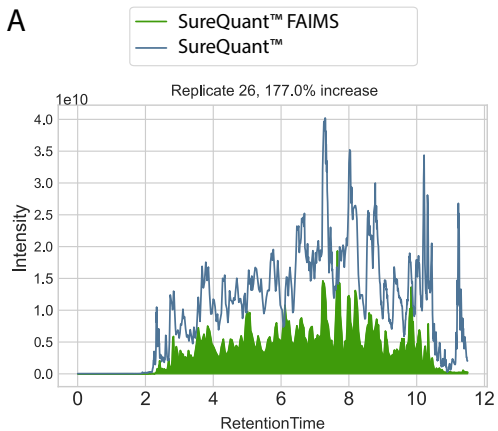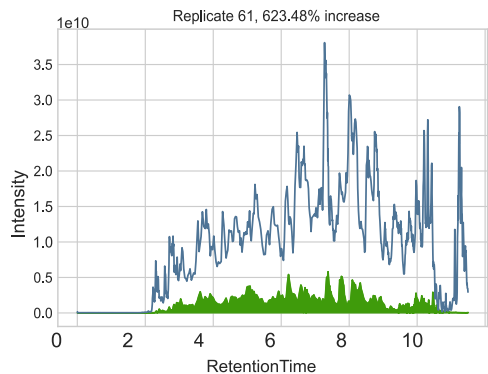

B

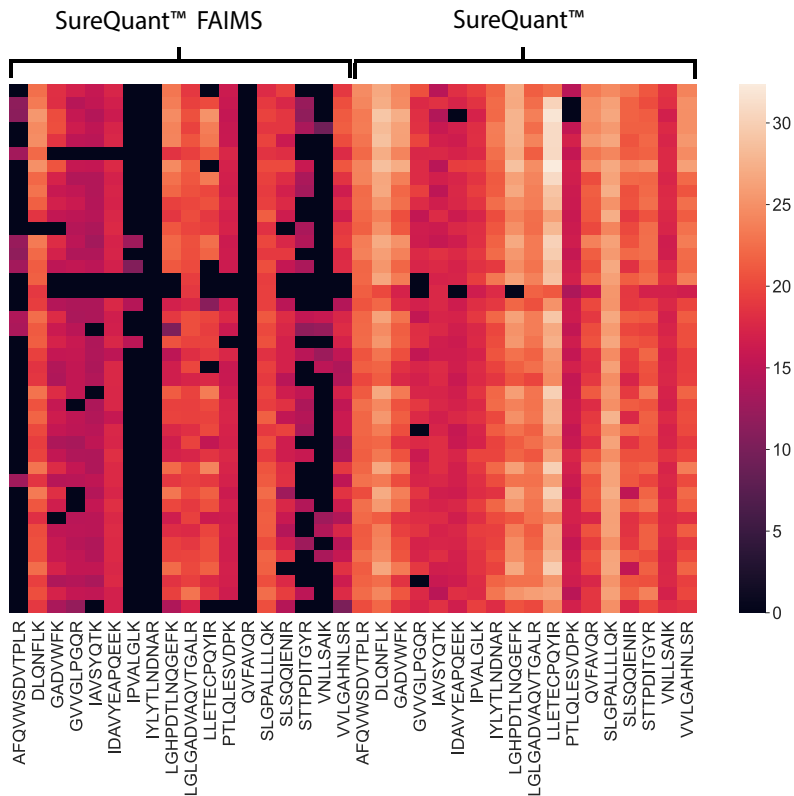

Supplement: Suppl_figure_1 — A, examples of TIC intensities between SureQuant and SureQuant with FAIMS. Addition of FAIMS during MS analysis results in reduced signal for the analytes measured. B, quantification values for the peptides monitored across SureQuan and SureQuant FAIMS analysis. Non detected or filtered precursors are indicated with black boxes. [file mmc1.pdf]

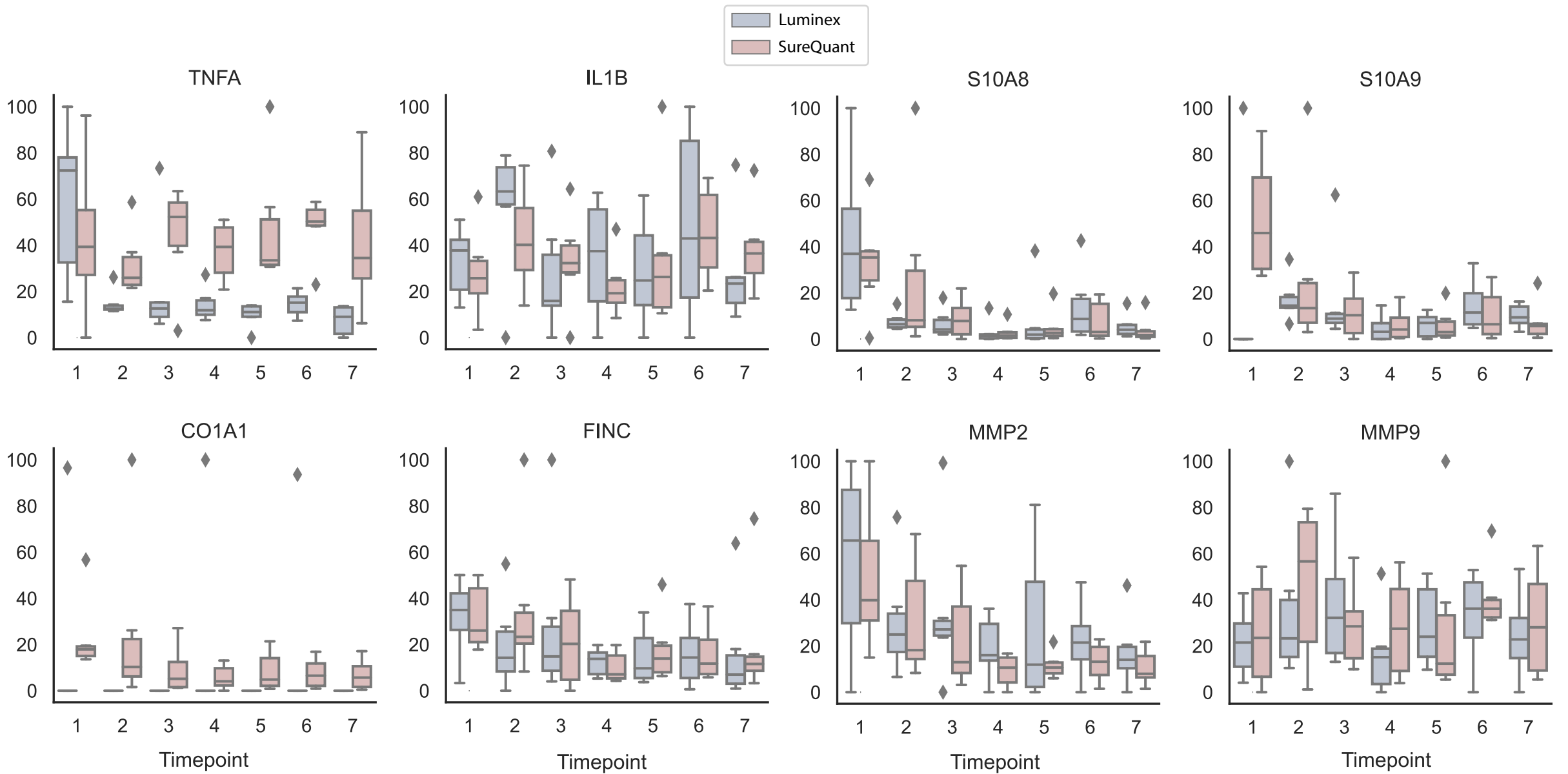

Supplement: Suppl_figure_2 — Comparison of quantification between Luminex and SureQuant measurements, for the 8 proteins measured. Collagen 1 A1 chain was not quantified in most samples with the Luminex technology due to values outside the standard curve. Values are normalized to the highest quantity observed. In SureQuant quantification, peptide intensitites are summed for protein quantification values. [file mmc2.pdf]
